# Interculturalism in the post-multicultural debate: a defence

**DOI:** 10.1186/s40878-017-0057-z

**Published:** 2017-09-04

**Authors:** Ricard Zapata-Barrero

**Affiliations:** GRITIM-UPF (Interdisciplinary Research Group on Immigration), Department of Social and Political Science, Universitat Pompeu Fabra, Ramon Trias Fargas, 25-27, 08005 Barcelona, Catalonia Spain

**Keywords:** Diversity, Interculturalism, Public policy, Xenophobia, Multiculturalism, National civic policy

## Abstract

The main purpose of this article is to formulate a defence of the emerging intercultural policy paradigm for the benefit of those who are still somewhat reluctant to accept its proper place within the current migration-related diversity policy debate. My defence will take two main lines of argumentation:

Firstly, I will state that the increasing intensity of the intercultural policy paradigm must be placed in the present-day post-multicultural period, which recognizes the strengths ​​of the multicultural policy paradigm but also the limits to its process for recognizing differences. The role played by the emerging national civic policy paradigm (a renovated version of assimilation), prioritizing duties before rights, will also be considered crucial to better contextualize interculturalism.

Secondly, I will try to identify the main distinctive features of interculturalism, which legitimize its proper place within the diversity debate today. Without rejecting rights-based and duties-based policy approaches, interculturalism places more emphasis on a contacts-based policy approach, aimed at fostering communication and relationships among people from different backgrounds, including national citizens. This approach focuses on common bonds rather than differences. It also views diversity as an advantage and a resource, and centres its policy goals on community cohesion and reframing a common public culture that places diversity within rather than outside the so-called Unity. In reviewing the current literature and the origins of the intercultural policy paradigm, I restate its contribution towards resolving current trends in transnationalism, changing identities, superdiversity and the rise of populist anti-immigrant parties. These are issues the old multicultural project has struggled to deal with, which has provoked the current disillusionment. Lastly, I will propose a research avenue to further consolidate interculturalism as a distinctive and legitimate policy approach.

Diversity management is lacking reference points after a backlash against multiculturalism (Vertovec & Wessendorf, 2010) and increasing support for xenophobic political parties, most of which are also Eurosceptic (Hartleb, 2011; Leconte, 2015; Chopin, 2015). The financial crisis has also forced many governments and administrations to cut back on budgets originally destined for immigration policies. Most of them are even claiming they must produce policies at zero cost (Scholten, Collett, & Petrovic, 2016), withdrawing or diverting initial specific policies regarding mainstreaming policies. This, together with the associated increases in competition for resources between host and migrant communities, is reducing solidarity (Kymlicka, 2016). The new context of superdiversity (Vertovec, 2007, 2014) and the fact that multiple identities and transnational practices are becoming the norm is evidence that we are entering a post-racial period where factors such as birth-origin and nationality do not necessarily drive diversity policies; other factors such as social class, gender, age, current legal situation and working conditions also come into play. The fact that the securitisation framework has penetrated most diversity-management thinking, preventing more open, cosmopolitan and humanistic policies towards newcomers and those who have already been living in reception societies for some time, and the fact that in recent years strong trends have emerged showing that second-generation migrants are strongly embracing radical outlooks, are signs of a very extreme situation for the core project of multicultural Europe (Triandafyllidou, Modood, & Zapata-Barrero, 2006). Brexit and the end of free movement for EU workers (Favell, 2014) also poses serious difficulties to maintain one of the markers of European identity, namely, European citizenship (Zapata-Barrero, 2016a), and certainly contributes to the need to reframe the European project (Triandafyllidou & Gropas, 2015). In such times of turmoil and in this context of a crisis of ideas, interculturalism may help light the way, enjoying as it does some traction in policy and political spheres, and some support from experts and in academic circles, indispensable conditions for being considered a policy paradigm.

This initial dynamic contextualization illustrates that we cannot understand the emergence of the intercultural policy paradigm (ICP) in migration and diversity studies from a static perspective. The ICP is the result of a historical process and the outcome of many factors that today reframe the migration-related diversity policy debate. The best way to focus this discussion is in terms of continuities and changes, and to approach it in terms of policy paradigm change and formation (Zapata-Barrero, 2017b). Against this backdrop I shall defend two key arguments.

Firstly, the emergence of the ICP must be placed in the current post-multicultural (post-M) period, which recognizes the strengths of the multicultural policy paradigm (MCP) in setting equality, power sharing and inclusion, but set limits to this process of recognition of differences. The role played by the emerging national civic policy paradigm (NCP) (a renovated and perhaps more inclusive version of the former assimilationist policy paradigm) in placing duties before rights will be seen as critical. This post-M period also illustrates an increasing academic awareness that casts doubt on the way the debate has been conducted in the past in terms of a Unity and Diversity nexus. This framework debate needs to be replaced together with its version that separates immigrants from national citizens for the creation of diversity policies, as this assumed separation has caused tensions, which, instead of solving issues, belong to the problems that need to be solved. This is how I place the old multicultural policy approach. New recognitions that we are in a super-diverse society, governed by increasing transnationalism in all its facets – complex multiple identities becoming more and more the norm – make it harder to encapsulate diversity issues in such outdated frameworks.

Secondly, I will show the main distinctive features and commonalities of interculturalism with the other two policy paradigms that today govern the post-M period, focusing particularly on the contrast with MCP, given the basic theme of this special issue. Without necessarily rejecting the rights-based and duties-based policy approaches, interculturalism places more emphasis on a contacts-based policy approach. Following some key dimensions of the policy-paradigm change debate, I will defend the position that the ICP is a new policy strand aiming to foster communication and relations among people with different backgrounds – including nationals – and to focus on common bonds rather than differences. It also has a particular view of diversity as an advantage and a resource, and its main normative policy drivers are community cohesion and a diversity-based common public culture. The specific features of its local and pragmatic policy origins – contrasting with the state-centred and universalist view of multicultural policies – will complete the picture. With these key distinctive features, I shall defend that in the current day and age – a time suffering feelings of failure and disenchantment with old diversity policy paradigms – interculturalism may well be the best interlocutor within the MCP and the NCP debates governing most of the current post-M scholarly discussions. I will end by proposing a research avenue to win further trust for interculturalism as a distinctive and legitimate policy paradigm approach, one that can help us overcome this failure mentality in which we appear to find ourselves.

## Continuities and change in the post-multicultural framework: rebooting the unity/diversity debate

The MCP has dominated recent decades, holding a monopoly over the narrative on how to reconcile Unity and Diversity, and essentially following the equality and human rights principles on diversity management, with a normative conception of justice in the background. We know that there are different perspectives on how each scholar focuses the diversity, equality and human rights interface (Laden & Owen, 2007; Bloemraad, 2007, 2015; Wise & Velayutham, 2009; Triandafyllidou, Modood, & Meer, 2011; Crowder, 2013; Mansouri & Muraca, 2014; Kivisto, 2016; Song, 2016). To summarize MCP’s nuclear core, its main project is the inclusion of immigrants into the mainstream by respecting their differences and recognizing their distinctive cultural practices, religions and languages. Economic distribution and political participation is also one of the main building blocks (Kymlicka, 2010). Recently some scholars have focused on the MCP in terms of indicators rather than principles (Levy, 2000, p. 125–160; Murphy, 2012; Banting & Kymlicka, 2013; Bloemraad & Wright, 2014; and even Vertovec, 2010), providing additional specific evidence-based structural and legal arrangements to ensure the non-alienation of specific groups. In such empirical studies multiculturalism has deployed most of its tools for the protection of rights, for the containment of exceptional cases within the mainstream public policy system, and legitimating specific policies basically in terms of funding, recognition and affirmative action. And a certain group-based approach has been dominant in the application of the principles, without incorporating a more critical view of what kind of cultures deserve recognition and under what terms.

Sharing this evidence-based approach and fully aware that times have changed, Kymlicka highlights some contextual factors that challenge the MCP. He illustrates, for instance, that:

existing theories of liberal multiculturalism presuppose, implicitly or explicitly, that state-minority relations are “desecuritized” – that is, the governance of state-minority relations is seen as an issue of social policy to be addressed through the normal democratic process of claims-making, consultation, and debate, not as an issue of state security that trumps normal democratic processes. (Kymlicka, 2015, p. 241)

He also signals that some of the conditions of multiculturalism are eroding:

Liberal multiculturalism, I would argue, was theorized for situations in which immigrants were seen as legally authorized, permanently settled, and presumptively loyal. In an age of securitization and super-diversity, these assumptions are put into question. Early theories of multiculturalism now seem at best incomplete, and at worst out-dated, resting on assumptions and preconditions that may no longer apply. (Kymlicka, 2015, p. 244)

As Kymlicka (2010) foresaw, the new historical phase that we are presently in is characterized by the fact that most of the multicultural criticism comes not from a far-right, anti-immigrant and nationalist discourse, but from inside multiculturalism. I consider myself within this trend. What Kymlicka was claiming is the need to reframe multiculturalism within a new context, something we can label, for want of a better term, as the *post-Multicultural era*. It is within this new phase that I would like to place the current emergence of the ICP.

I am aware that the term ‘post-M’ could contribute to confusion rather than clarification if conceptually it is not well defined (Ley, 2005; Bradley, 2013; Gozdecka, Ercan, & Kmak, 2014; Matejskova & Antonsich, 2015). Primarily it involves a need to merge the Unity/Diversity dimensions that have featured throughout the diversity policy debates of recent decades, incorporating the contextual factors framing the new times of turmoil that were described herein at the outset. This basically means that today, where a plurality of identities and transnational minds has become the norm in our super-diverse societies, where diversity is seeking to enter mainstream policies, to argue about how to deal with diversity-related socio-economic and power relations in terms of majority-Unity-us and minority-Diversity-others is to condemn the new reality to distortion, fueling the arguments of its detractors. This taken-for-granted analytical framework of conducting research is causing nowadays serious limits in developing knowledge in migration studies. It implies also that we are in a process of policy paradigm change, where most of the main pillars of the MCP remain, but with a rising awareness that the recognition of differences following the equality principle cannot be defended without requiring a justification. And legitimate proofs cannot come from liberal and democratic principles (the initial project of multicultural citizenship) alone, but need to be formulated essentially in terms of community cohesion and a new diversity-based common public culture. Also belonging to this trend is the growing conviction that in settings of complex diversity, tolerance needs to be limited (Zapata-Barrero & Triandafyllidou, 2012; Dobbernack & Modood, 2013). This debate certainly comes in a context where the MCP is at one of its lowest moments (Lewis, 2014), under suspicion of having promoted segregation rather than union, of giving rise to ethnic conflicts rather than a common culture, of struggling to offer grounds for community cohesion and social capital (Cantle, 2008), and even of legitimating affirmative actions. Today, there is a growing awareness that multicultural policies have fuelled far-right xenophobic political parties. In Germany in October 2010 and in the United Kingdom in February 2011, political leaders have also promoted this argument of state multicultural failure, a backlash against – or even the ‘death’ of – the multicultural paradigm, provoking deep public discussion across Europe (Daily Mail Reporter, 2011).

This growing concern in Europe over the rise of populist anti-immigrant parties and anti-Islamification narratives cannot be disconnected from the disenchantment with multiculturalism. What is new today is the electoral support that some of these political parties have gained in some countries, significantly providing real alternatives for power and provoking a *contagion effect* to mainstream political parties. The recent general elections in France (May 2017) also demonstrated that these parties, after an initial period of conquest, seem to have established themselves in the mainstream political system. This has even meant that governments have changed their courses of action, incorporating anti-immigration measures into their strategies for managing diversity (Ferruh, 2012), a situation that has been aggravated by contradictions within the immigration politics of the liberal states forced by theses contextual restraints (Hampshire, 2013). What is specific to the debate on growing radicalism against diversity is that it uses most of the basic normative premises that legitimate the MCP, and in this sense it is a scholarly forum that must be taken seriously by strong defenders of liberal democratic principles and human rights. It would be lacking in historical insight and academically irresponsible to misinterpret the elite discourses that have framed most of the public debate in Europe in recent years. The ‘muscular’ defence of liberal democratic principles, to borrow the words of former British Prime Minister D. Cameron, has provoked an array of criticisms; however, there is a clear purpose to address the multicultural question in terms of limits:

Under the doctrine of state multiculturalism, we have encouraged different cultures to live separate lives, apart from each other and apart from the mainstream. We’ve failed to provide a vision of society to which they feel they want to belong. We’ve even tolerated these segregated communities behaving in ways that run completely counter to our values. (Cameron, 2011).

This means that immigrants must, at minimum, acquire the language of the host country, and learn about its history, norms and institutions. And it entails the introduction of written citizenship tests and loyalty oaths. Implicitly if not explicitly, national civic integration is presented as the only tool to limit what we may call a *boundless multiculturalism.*

The backlash against multiculturalism does not express a problem with culture, but rather with its excess, including in its most extreme form the recognition of illiberal values and a lack of human rights’ protection. For instance, Banting and Kymlicka (2006) already pointed to a relationship between anti-multiculturalism and illiberal practices perceived within the kinds of cultures being accommodated. More precisely, they warned that “it is very difficult to get public support for multiculturalism policies if the groups that are the main beneficiaries of these policies are perceived to be carriers of illiberal cultural in order to maintain these practices” (Banting & Kymlicka, 2006, p. 54). Of course this zero-sum way of seeing multiculturalism and its limits, as it has been first articulated, among others, by Joppke (2004, 2008), has to be relativized. The new context is such that, as Vertovec (2010) has notably stressed, “No politician wants to be associated with the M-word”. To be post-M then does not mean being anti-M, but rather incorporating within the multicultural policy project the awareness that not everything coming from other cultures can be accepted without a critical mindset.

The national civic turn belongs to this post-M era.^1^ Why does this post-M framework emphasize the view of considering national identity as a friend rather than a foe? Because there is a certain shared view that the MCP has exaggerated the rights-based approach to the detriment of duties. And these duties towards immigrants must also be placed at the same level of policy consideration, because they can help to regulate the excessive recognition to certain cultures, and limit illiberal practices contravening human rights. In practical terms, the duties-based approach calls for development of the means to ensure civic practices and citizenship as well as a minimum level of competence in the national language and a minimum level of knowledge about the country’s history and society. In normative terms, it seeks to ensure a minimum threshold for living together in a common public culture. It is true that this civic turn can have many readings, depending on how one sets this minimum threshold and if one makes it voluntary or compulsory. In European terms, taking care not to erode Unity by being “too diverse”, to use Goodhart’s (2004) terms, means reevaluating national identity, language and democratic liberal values as limiters rather than promoters of multiculturalism. There is however in this new civic national narrative a problem, which was already visible in the multicultural approach: they both still consider diversity as “the other” that is separated from the mainstream, instead of placing diversity *within* the mainstream. The question today is no longer how to live *with* diversity but how to live *in* diversity (Antonsich, 2016, p. 470). The growing diversity scenarios compounding our societies today are new for everybody, whether their origins are Filipino, Pakistani. Moroccan, Chinese, Ecuadorian, French, German, Hungarian or Italian. There is a general desire to build an alternative to the extremist narrative, and neither the MCP nor the NCP narratives that are dominating this post-multicultural period provide us with enough convincing arguments that bridge Unity and Diversity. I would even say that this post-M period places multiculturalism under suspicion of being part of the problem needing to be dealt with.

This post-M era also means we are entering a post-racial period, as those who oppose multiculturalism see it as having been imposed by racial and ethnic minorities whose demands for recognition were prioritized over all other concerns. The unease surrounding multiculturalism, which has led governments across Europe not only to ban hijabs and burkas but also to install citizenship testing and promote ‘national values’ (Lentin, 2014, p. 1272), has less to do with multicultural policies and more to do with fragmentation and the loss of a common public culture. It is a kind of fusing of the Unity and Diversity agendas, or, as King described this post-M as a wide acknowledgement of group distinctions combined with a state struggle to ensure that government policies do not accentuate hierarchical divisions between groups based on race, ethnicity and national background; a struggle rich in historical connotations that can no longer presume a teleological narrative towards melting-pot individualism (King, 2005, p. 122). This claim that Unity also needs to be respected and recognized within Diversity is gaining support from a number of scholars. In terms of rights and duties, this post-M period seeks to focus the debate on the best way to reach some sort of Rawlsian reflective equilibrium, where duties limit the rights-based calls for the recognition of differences.

Some scholars now acknowledge the defensive role that multicultural theory must play in academic and public circles (Bradley, 2013). But the form of logic used in this defence must be questioned: it is professed that the critics of multiculturalism are wrong, that they deform and stigmatize multicultural ideology. This trend has even been made personal, with unjust criticisms purported, for instance, by Stokke and Lybæk (2016), when they even accuse interculturalists in general and Ted Cantle (2017) in particular of representing the “white majority position”, as if most of the defenders of multiculturalism with non-national backgrounds were representatives of multiculturalism but not assimilationism. This *elitist multiculturalism* is, in my view, the wrong path to follow. We are entering a period where most scholars are becoming aware that there is a need to combine many policy approaches with, one could say, an intercultural mindset. In terms of Unity and Diversity management, not everything is white or black, majority or minority (Modood, 2016). The new policy perspectives are not incommensurable or conflictive such that the implementation of one automatically nullifies the possibility of supplementing it with the other.

In this epistemological context, the added value of this post-M framework is not only that it officializes the need to limit the former *boundless multiculturalism* narrative through more civic national values, but that interculturalism becomes a kind of mediator between the two, placing emphasis on the communicative aspect within Diversity, which also belongs to Unity. As I do not have space to devote even a short section to the national civic turn or to the duties-based approach to diversity, I will simply say that this “civic zeitgeist” describes a set of practices including integration contracts, classes, citizenship tests and ceremonies, acquisition of the language of the host country, and learning about its history, norms and institutions (Meer, Mouritsen, Faas, & De Witte 2015). This NCP may be said to have the mythical dual faces of Janus, since it cannot be interpreted only as part of a more or less hidden nationalistic assimilation agenda, but must also be seen as a policy narrative ensuring equal opportunities and a minimum cultural capital for the development of social capacities in the host society. It can also be seen as an instrument to facilitate a sense of mutual belonging, contact and interaction. This NCP is also becoming an example of a multi-level policy (although this needs deeper analysis), since it acts both at the level of a welcoming policy, basically administered by cities, and at the level of citizenship acquisition, which is the exclusive remit of states. My view is that in spite of some multiculturalists claiming compatibility, questions posed by one of the most constant critics of multiculturalism (Joppke, 2004) remain unanswered. This is why the debate cannot dismiss the most radical approach of the civic turn, which fundamentally places duties as a condition for allocating rights. This argument exists in many policymakers’ and politicians’ minds, and in its radical form (that is “no rights without duties”) it not only attracts right-wing and populist anti-immigrant political parties, but also social-democrat political parties who see that these policy narratives, together with the “welfare chauvinism” narratives, may help them to win over more of their electorate.

## The intercultural policy: going with and beyond the rights-based and duties-based policy approaches

The ICP starts out with a claim for greater dialogue, considering this to be something lacking in the two former paradigms. In addition to the rights-based and the duties-based approaches, there is a need to focus on contact between people from different backgrounds, including nationals. Interculturalism does not seek ranking as the best way to deal with the accommodation of diversity, but rather to emphasize the need to focus on something other approaches seem to have taken for granted and which does not automatically occur if there is not a policy to target it specifically. This contacts-based approach is seen as an integration policy (Guidikova, 2015), as fostering intercultural citizenship (Zapata-Barrero, 2016b), and consequently it also needs to be seen as an important driver for a socialization process, of culture-making (Sarmento, 2014, p. 615). It takes its starting point from Allport’s (1954) contact hypothesis, which says that contact and sharing promote mutual acceptance under conditions of equality, and initiate a process of prejudice redution and knowledge formation. Through contacts, people can acquire what civic integration claims to achieve – knowledge and mutual understanding – and what multicultural integration also seeks to obtain – the combating of diversity-related discrimination and inequalities. It also allows people from different backgrounds, including national citizens, the same opportunities in society. Intercultural policies are therefore essentially seen as an anti-racist tool.^2^ It takes seriously the whole formulation of the well-accepted contact hypothesis: "Prejudice (unless deeply rooted in the character structure of the individual) may be reduced by equal status contact between majority and minority groups in the pursuit of common goals. The effect is greatly enhanced if this contact is sanctioned by institutional supports (i.e., by law, custom or local atmosphere), and provided it is of a sort that leads to the perception of common interests and common humanity between members of the two groups" (Allport, 1954, p. 281). This *anti-discrimination promotion* is a fundamental element of the ICP since it focuses on the factors which hinder contact zones. There are contextual, legal, institutional and structural factors that reduce people’s motivation to interact and even build walls of separation between them based on misinterpretations of differences. Here we take into account legal frameworks concerning voting rights for foreigners and naturalization policies, as well as socio-economic opportunity gaps among citizens, where differences become the factor behind reduced contact. Anti-discrimination promotion also includes tackling disadvantage, as it is hard to see how the ICP can continue over time if one or more sectors of society are so unequal that people are led to believe they have no real stake in that society.

We can say the ICP is a technique for bridging differences and creating bonds and social capital. That is, it promotes relations between people who share certain characteristics (bonds), as well as relations between individuals from different backgrounds (promoting interaction between people across different religions, languages, etc.) who are predisposed to respecting others’ differences (Gruescu & Menne, 2010, p. 10). It is a way, then, to avoid the confinement and segregation of people, which may condemn them to a timeless social exclusion. The *descriptive definition of interculturalism* that follows relates to a wide range of different types of contact, from circumstantial and sporadic communication in the marketplace or out of schools among parents from different backgrounds, to inter-personal dialogue and even interaction, which implies the sharing of a common project, or even inter-dependence, where to reach an expectation or purpose people are reliant upon others’ actions. In all likelihood a deeper examination of the gradual contact processes still needs to be carried out by interculturalists, but the fact that contacts become a driver in policy creation becomes a key premise.

Most of the premises for legitimacy supporting the intercultural policy narrative (as discussed further on) come precisely in the added value of these communicative practices and the justification of how policy intervention to foster relations can benefit society. But we cannot perceive interculturalism as something that should be compulsory, as it were a perfectionist philosophy. If people do not want to communicate, we cannot force them. The problem arises when we notice that, most of the time, people are not in contact because there are certain constraints that take the form of prejudgements and stereotypes. It is here that many programmes aimed at combatting rumours, prejudices and negative perceptions towards diversity are in expansion in Europe (see Council of Europe, 2014). Consequently, we must be aware that while contact is fundamental to interculturalism, it also needs to be supplemented by a positive narrative at the societal level to support and sustain the beneficial impact (political leaders’ narratives, the media, schools, etc.).

Of course, promoting contact also has dimensions relating to social class, but we are pointing here to negative perceptions and preconceptions. At this point, this policy takes an instrumental form, focusing on the conditions for communication, and it is here that interculturalism meets the multicultural and civic integration policies, considered as necessary conditions for contact. For instance, Meer and Modood (2012) argue that to allow communication, there are certain equality and power sharing requirements, or, as Levrau and Loobuyck (2013, p. 622) highlight, the fact that the NCP assumes the need for a minimum level of shared language and public culture, setting the basis for communication (see also Barrett, 2013). Interculturalism begins then when the multicultural and national civic policies have developed all their potential, not instead of them, against them or before them. Interculturalism does not see the MCP and the NCP in disjunctive terms (either one or the other), but rather as complementary paradigms. Without a certain degree of recognition of rights and fulfilment of duties, contacts can become difficult.

Let me now position interculturalism as a new policy narrative inviting a process of policy paradigm change. I will borrow some frameworks that originate from current public policy debates.^3^

## Intercultural narrative policy: origins and first premises

The origins of the intercultural policy narrative stem, like those of the multicultural policy paradigm, from Canada. It developed as a 1980s reaction to the Canadian multicultural policy announced in the 1970s, which placed Québécois identity in the same basket of diversity as the indigenous population and immigrants (the three basic dimensions of multiculturalism that had penetrated the debate, Kymlicka, 1995) and was basically seen as detrimental to the survival of the French language. It claimed that the interrelation between the minority and the majority (Québécois culture) must be the centre of the negotiation, and then directly placed the dialogue between Unity and Diversity as the main framework to legitimize policies towards immigrants. This intercultural approach was seen, however, in the context of *multiple diversity*, that is where two dynamics of diversity interact (the national demands of Quebec and the cultural demands of immigrants)^4^ and this policy was considered an instrument to ensure the survival of the Québécois national identity. Many scholars have articulated this policy philosophy (Gagnon & Iacovino, 2016; Labelle & Rocher, 2009) but the one who without doubt has been the most influential is Bouchard (2015), also largely responsible for the Bouchard-Taylor report (*Building the Future: A Time for reconciliation*, Bouchard & Taylor, 2008), the result of an scholarly and public open debate that laid the foundations of this intercultural philosophy. This vertical view of contact between Unity and Diversity, understood in terms of a power relation between the majority and minorities, constitutes the ‘Québécois’ view of interculturalism and develops a new contract theory putting emphasis on some sort of reflective equilibrium between the rights of migrants and their duties to respect the Québécois culture and language (Zapata-Barrero, 2015a). In my view it is certainly unwise to try to decontextualize this Québécois view of interculturalism by moving this debate to Europe in terms of majority and minority communicative trends, as Modood, (2014) has recently proposed.

In Europe, interculturalism has a different origin. It does not arise in a contextual debate on *multiple diversity*. It is a policy narrative that in practice had probably been in existence earlier, but not as a concept giving its name to a public policy paradigm. Using the terminology of linguistics, one could say that the *notion* existed before the *concept*. I have shown, for instance, that in 1997 the city of Barcelona opted to call its policy ‘intercultural’ as a result of dissatisfaction with the existing multicultural/assimilationist models in Europe (Zapata-Barrero, 2017a). Essentially, this was justified because multiculturalism was seen to be distant from the mainstream policy and assimilationism did not respect legitimate cultural practices of certain groups. From the very beginning, a focus on promoting contact rather than separation, on working towards immigrants’ inclusion as much as possible and on devising policies concentrating on immigrants within the basic mainstream structure of public services was put forward as an integration policy that responded better to current views. This intercultural policy was also theorized in other parts of Europe as an inclusion policy with the central purpose of preventing socio-economic exclusion.^5^ The first theoretical articulation came from experts (not from academia, as was the case with MCP) Wood and Landry (2008), whose urban intercultural philosophy influenced the intercultural city programme of the Council of Europe, launched in 2008 during the European Year of Intercultural Dialogue. P. Wood inspired the aptly named intercultural manifesto, with the *White Paper on Intercultural Dialogue. Living Together as Equals in Dignity* (2008). Since then, a practical step-by-step guide has served as the main document to frame the first internal debates among local policy makers, and an intercultural city index (ICC) is being applied across Europe and further afield (Mexico, Montreal, Tokyo) to benchmark its implementation.

This ICC gives us valuable and primary information on how the ICP is defined through 10 main dimensions: Assessment of city functions “through an intercultural lens” (education, the public domain, housing and neighbourhoods, public services and civic administration, business and the economy, sport and the arts); Urban safety; Mediation and conflict resolution; Languages; Media strategy; Establishing an international policy for the city; Evidence-based approach; Intercultural awareness training; Welcoming newcomers; and Intercultural governance (which includes participation and representation). These dimensions constitute a comprehensive range of different areas of intervention for ensuring the conditions to foster relations among people from different backgrounds, including national citizens. Nowadays, the intercultural cities programme of the Council of Europe has more than 100 cities working together, including national networks in Spain, Italy, Norway, Ukraine and Portugal, sharing know-how, policy experiences and good practices relating to the implementation of intercultural policies.

Coming mainly from urban, management and business studies, but also from social psychology, the first promoters brought with them a different concept of diversity, which was not considered by either multiculturalist or civic nationalists: the concept of *diversity advantage.* 6 Here is not the place for analytical discussion of the many facets of diversity (Vertovec, 2014, 2015), but what is crucial is to highlight that this notion of diversity as a potential resource and a source of opportunities, which needs to be managed to make full use of its advantages, is path-breaking in current post-M debates, and absent in multiculturalism from the very beginning.

The ICP in Europe takes this particular conception of diversity as a potential benefit for society, and it has been interpreted as a policy strategy to promote these advantages. This specific instrumental focus has an individual and a socio-economic dimension. At the individual level, we may be able to find a related concept of diversity in migration studies with the seminal article by Faist (2009), who claims that diversity must try to go beyond the rights-based approach and be seen as an individual competence, but Faist does not go so far as to enter the intercultural philosophy debate. The fact is that the ICP sees individuals as holders of competences that need to be promoted. It is at the level of such competences that the opportunity structure and equality principles can be reassessed. From urban studies, this approach emphasizes the view that diversity is a community asset and a collective resource, since it is assumed that optimizing diversity increases social and political benefits (Wood & Landry, 2008). An immigrant has extra competences and skills relating to social and cultural capital, such as language, differentiated cultural registers, and culture-specific worldviews and knowledge. The managerial economist Page (2007) is often quoted in the literature of this emerging field, as he shows that in a problem-solving situation, diverse groups have better tools and resources to give a variety of perspectives than a homogeneous group. At this individual level, we also know from the seminal influent work of Berry (2013) that interculturalism is seen as the most appropriate tool to promote creativity in society, and some of its followers are trying to strengthen the distinctive assets of inter-group relations (Howarth & Andreouli, 2013), such as trust, mutual-knowledge and prejudice reduction. As regards the socio-economic dimension, this diversity advantage approach is also supported by global business studies (Zachary, 2003), where the focus is on the economic benefits of diversity. Applied to society, this means that diversity can be seen as a driver of social and economic development. This line of discussion connects with other studies that follow the traditional view of the economic benefits of immigration (Borjas, 1995). The link between diversity and economic performance is already producing interesting work and contributing to the consolidation of the ICP (see Alesina & Ferrara, 2005; Janssens, Pinelli, Reymen, & Wallman, 2009; Bellini, Ottaviano, Pinelli, & Prarolo, 2009; Müller, Wagner, & Kunz, 2011; Wagner, 2015). Yet, a number of studies on the intercultural approach include discussions about diversity’s impact on growth, productivity and employment (Khovanova-Rubicondo & Pinelli, 2012); about governance structures and processes (see for instance Zapata-Barrero, 2016c); about urban planning (see for instance, Wood, 2015); about housing and neighbourhood policies; and about security and policing policies. The awareness that the key to giving some continuity and permanence to this ICP must come from these evidence-based arguments has been there from the very beginning, with the knowledge that probably most of the MCP’s key legitimacy problems have their roots in these empirical shortcomings, having first been thought up in academia without being properly tested. This gap between policy and implementation has also been there since the very beginning (Lægaard, 2016).

The concept of diversity, as articulated by the Council of Europe (2011), is grounded in this interlink between business, social psychology and urban studies. This means re-designing institutions and policies in all fields to treat diversity as a potential resource and a public good that needs to be distributed, and not as a nuisance to be contained. In practice, this diversity management is excellent for providing equal opportunities for education, employment, entrepreneurship, holding civil office, etc. (Wood & Landry, 2008; Guidikova, 2015).

Ted Cantle’s writings are another source of the ICP in Europe, illustrating a less constructivist focus and drawing a much more social and cosmopolitan strand of interculturalism. It also has a particular view of diversity, probably close to the preventive concerns described in Putnam’s (2007) seminal work. That is, diversity without policy intervention can be a source of conflicts and can increase the socio-economic disadvantages of diverse people. The notion of conflict related to diversity has to be understood in a broad sense encompassing racism, poverty and social exclusion (Cantle, 2012, p. 102). In this sense, we can also see that there is a certain preventive dimension in the ICP. This second more preventive view of diversity has been articulated again by another expert: Cantle (2012). He was responsible for a report on the British government’s concern for local riots stemming from social unrest in northern towns in August 2011 (in Bradford, Burnley and Oldham). These events directly linked social conflicts with the failure of British multicultural policy. His book *Community Cohesion* (Cantle, 2008) articulated these ideas against the MCP, and argued against the promotion of ‘parallel lives’ between communities that had little in common and no contact with each other. The debate between multiculturalism and interculturalism in Britain quickly took this contextual framework, and featured the contrasting views of T. Modood and T. Cantle on how to understand the place of diversity and culture in integration policies (for a good introduction to the debate, see Antonsich, 2016).

The idea of ‘community cohesion’ comes then to the debate, as a new normative driver together with the diversity advantage promoted by Wood and Landry, and the view of interculturalism as a tool for negotiation between Diversity and Unity, *à la* Bouchard. The central claim of the ICP here is that there is a need to go beyond the ethnicization of politics and the very concrete concept of culture related to national identity and race. This post-national and post-racial view of culture is certainly a direct critique of the MCP’s core assumptions, and allows us to centre the policy on common bonds, which must prevail over differences as a premise on which to formulate policies. Promoters of the ICP are fully aware that shared practices and relations can be constrained by inequality and asymmetrical power relations, and by the lack of a minimum level of common public culture. It is probably at this point that interculturalism shows its most demanding side, requiring the appropriate conditions for inter-personal relations and mitigation of the risk that contact zones could become conflict zones, particularly in vulnerable areas where tensions between communities prevail.^7^ Probably the added value of the ICP is that in promoting contact between people from different backgrounds it helps also to develop intercultural values such as trust, common understanding and what I have called elsewhere a *culture of diversity*, which essentially means going beyond the simple fact that the current contexts of cities are diverse, in order to discuss how diversity is being incorporated into public and civic culture, at the level of both institutional structures and routines. This *culture of diversity* can be seen as a by-product of what in the recent work of Matejskova and Antonsich (2015, p. 3) is called governance *through* diversity rather than governance *of* diversity. The *through* indicates that diversity does not exist apart from politics, but needs to be incorporated within governance, the incorporation of diversity in political parties probably being the first step (Zapata-Barrero, Dähnke, & Markard, 2018) with further research required for other pillars of society (schools, police, administration, etc.).

An example of an intercultural concern when requesting citizenship tests could be whether diversity is incorporated as a common value to be respected or still treated as something that falls outside Unity? This is why I define this *culture of diversity* through three main standards: Diversity recognition, Diversity participation and Diversity management (Zapata-Barrero, 2014, p. 68). In this claim of incorporating diversity in all spheres of the society, the ICP can also fight to include diversity within the cherished notion of “common value” articulating the civic national policy narrative. The civic integration policy speaks about a culture of citizenship (Mouritsen, 2009) rather than a culture of diversity, and then seems still to place diversity outside rather than inside mainstream society.

Compared to the MCP and the NCP, what do these three normative parameters of the ICP tell us in relation to setting the premises for a policy paradigm change? First, by virtue of its origins, the European view of interculturalism can be seen as a *policy rebellion of cities* against the state domination of policy in recent decades. The multicultural and civic integration models were thought out essentially at the state level and have rarely considered multi-level perspectives in the implementation of policies. This state-centric view of diversity management probably explains why their defenders propose rights-based and duties-based approaches to diversity, since they assume the state is the first institution addressing the diversity issues and framing whatever policy at whatever level of governance. Together with a different view of diversity, the MCP’s and NCP’s national-state dependency methodology is probably one of the first substantial differences when we compare them with the ICP. Cities are now organizing welcoming events for newcomers as a way to provide knowledge about the host society and the city, and they also offer language courses as part of the city’s public assistance. In contrast, multicultural policies are also developed at the city level, but always under an administrative decentralization process beginning at the state level.

To this local origin we can add the ICP’s origins in expertise. Here we can say that the research/policy nexus debate can help us to distinguish the contacts-based policy paradigm (interculturalism) from the rights-based (multiculturalism) and the duties-based (national civic) approaches to integration. The MCP comes from academia and has therefore an intellectual origin; it is a product of the “laboratory” albeit without too much testing at the beginning, and is deontic, universalist and rationalist. It is close to what Scholten (2011) labels an ‘enlightenment model’ within the research/policy nexus, where academic arguments predominate over policy arguments.

The ICP is an evidence-based policy and, in Scholten’s terms, is much closer to an engineering model, where the primacy is in the policy, which informs academic arguments. It is based on strategies to achieve the normative ends of diversity advantage, community cohesion and a diversity-based common public culture. The ICP has a much more teleological dimension than multiculturalism. This origin provides interculturalism with two main strengths: *proximity*, as it primarily promotes face-to-face relations and develops most of its policies at the micro-level (Zapata-Barrero, 2015b, p. 187), and *pragmatism*, because action and practice prevail over any preconception of ideal justice or equality – “to the extent that less emphasis is placed on form and culture, and more on the subject that acts and therefore interacts” (Abdallah-Pretceille, 2006, p. 480). The ICP’s primary concerns are not about abstract or universal notions of justice or rights and goods in the context of diversity, as may be the case with the MCP, but about a society that takes advantage of the resource that diversity offers while also ensuring community cohesion. This *social ecological background to diversity* is totally absent in both the MCP and the NCP.

These local and policy origins probably explain why one of the ICP’s main areas of action is the public sphere, city spaces being a perfect example. The concern over how to manage contact in public spaces has been one of the areas neglected by multiculturalists. The policy focus on shared spaces (Wood, 2015; Cantle, 2016) is understood to cover the main areas where face-to-face communication between people from different backgrounds arises: in community gardens, libraries, public amenities, festivals and neighbourhood spaces, as reported by Bagwell, Evans, Witting, and Worpole (2012). Again, this is new evidence that in its right-based understanding of recognizing difference and its initial concern of making multicultural claims that are compatible with liberal democracy, multiculturalism has been formulated from a state perspective rather than a city perspective. As far as I am aware, the multicultural literature has not yet deployed any lines of research on how to deal with diversity in public spaces, the “contact zone” par excellence.

Finally, we can also add another particular facet of the ICP: the fact it can attract many types of governments and political parties and show how it is *non-ideological.* This means that the ICP, when incorporated as a city project for managing diversity, “resists” ideological variations in political governments, and is colour-blind from an ideological point of view (as is the case for most intercultural cities participating in the Council of Europe’s ICC). This has also been seen when analysing the intercultural governance of the Spanish network of intercultural cities (see Zapata-Barrero, 2016c). These features certainly explain its political attraction and territorial expansion in Europe.

## Summarizing the place of interculturalism in the post-M framework debate and beyond

Kymlicka recently proposed a new framework for discussion linking solidarity and diversity, arguing there is a trend in the debate that says the increase in immigration and the multicultural policies it often gives rise to have weakened the sense of national solidarity. This national argument within the multicultural debate has also been a focus of attention for Uberoi (2008), who argued that multiculturalism can change national identity. This creates a potential “progressive’s dilemma”, forcing a choice between solidarity and diversity. Behind this focus there is the premise – a “*corroding effect”* in the words of Banting and Kymlicka (2006) – that “multiculturalism policies are said to erode solidarity because they emphasize differences between citizens, rather than commonalities” (p. 11).

To my knowledge, the MCP narrative has never formulated a critical interpretative framework regarding the way culturally homogeneous states categorize diversity dynamics. The intercultural argument is that we cannot impose the majoritarian understanding of diversity categories upon others. Ethnicity is self-ascribed, flexible and cannot be imposed by those with the power to define diversity categories. The ICP narrative reacts against the ethnicization of people. This substantial distance from the MCP narrative in the domain of ethnicity, nationalism and race is likewise a departure from what Brubaker (2002) calls ‘groupism’, namely, “the tendency to treat ethnic groups, nations and races as substantial entities to which interests and agency can be attributed” (p. 164). The summary of these arguments is clear. The transnational reality in which most people live today tells us that mostly birthplace and/or nationality do not determine public identities. To ask someone where he/she is born with the purpose of gaining an initial idea of what public identity he/she holds is not as self-evident as it was in the past. Several studies working on transnational and complex identities as an empirical category show us that transnationalism and people’s growing mobility are currently pluralizing our identities and our self-national and cultural adscriptions (Favell, 2014). This is now the rule, one which needs to be incorporated into the current theoretical policy frameworks and migration studies.

This leads us to argue that interculturalism also calls for reassessment of what we may call the “*immigrant/citizenship divide”*, which has dominated the diversity debate in migration studies. What interests me in this divide is the consequence of always reproducing a certain discourse where “we” national citizens are not the subjects of diversity policies: the MCP (and more explicitly the NCP) has always taken for granted that *“Diversity is the others”.* In the policy-making process, there is a division of the population between citizens and non-citizens, nationals and non-nationals, immigrants and citizens. This migration focus has the effect of reproducing a certain power relation between majority-national citizens and minority-ethnic citizens or immigrants, a relation that fails to create bridges between these two sets of people. Instead, this framework reinforces the idea of separate categories of people, just as diversity policies have been mainly directed towards one part of the population, be they called immigrants, non-nationals, ethnic minorities, or a range of other conceptualizations in different countries and contexts. Today, in a superdiversity context, in a scenario where second and third generations of migrants live in Europe, where the only attachment to their society of origin comes from their parents (for instance, Crul, Schneider, & Lelie, 2012), most so-called citizens have an immigrant background, and consequently, this division of the population that probably made sense in earlier stages of the migration process is now very difficult to sustain. This assumption therefore needs to be revised.

Keeping this focus, I argue that a mainstreaming policy dismantles this narrative framework by incorporating the entire population (immigrants and citizens) into the policy focus. In targeting the broad population and incorporating diversity concerns within the general public policy focus, the ICP features all the main dimensions of a mainstreaming policy and also seeks to be incorporated into policymaking at all city levels and in all departments (Scholten & Van Breugel, 2018). The final goal is to create public services that are attuned to the needs of the whole population, regardless of their background. This mainstreaming policy has also been recently defined as an effort to reach people with a migration background through needs-based social programming and policies that also target the general population (Collet & Petrovic, 2014, p. 2).

This is pertinent to such a degree that maybe we need to abandon immigration policy as a policy directed only at migrants, and instead speak about *mainstreaming intercultural policy*, which would include all people in the scope of diversity policies. Moreover, in the context of the recent financial crisis, together with the post-M context of an ideological crisis in the MCP, there is certainly a contextual factor favouring the *elective affinity* between interculturalism and mainstreaming (Zapata-Barrero, 2017c), to the point that we can say today that mainstreaming is a distinctive feature of the intercultural policy philosophy. I would even say that this interface provides interculturalism with a powerful policy tool, solving most of policymakers’ concerns regarding the MCP. As a result, this *mainstreaming* ICP designates a public-policy philosophy that emphasizes both the significance of promoting contact (the core concept of interculturalism) in all spheres of public life and basic structures of society and the importance of all the components of the diversity dynamics, including nationals and citizens (the core concept of mainstreaming).

Elsewhere I have referred to these taken-for-granted policy frameworks as *multicultural idols* that have dominated our understanding of diversity management and the way we have produced knowledge about diversity (Zapata-Barrero, 2017a). This perspective could invite us to explore new directions in diversity debates.

## Concluding considerations: new directions in the intercultural policy research agenda

The current day and age has seen immigration and human mobility become representative of globalization, with an inherent lack of control over boundaries and impacts on economics and welfare. With our current interpretative frameworks, we usually see these phenomena meet with opposition, both because of the diversity they brings and because they falls prey to the nationalist agenda. In this context, interculturalism can help generate answers where a *boundless multiculturalism* or *rigid national civism* may have difficulties. This is probably one reason why the ICP can be seen to represent a challenge to both policy paradigms. Rootless cosmopolitan global citizens are despised by nationalists as much as they are by boundless multiculturalists. The post-M period where the diversity debate lays, illustrates that European societies have fallen to some sort of vicious circle. In the age of populism, to use Kymlicka’s words (Kymlicka, 2016), multicultural master narratives nurture anti-immigrant arguments and feelings, or even radical views of national civic integration, ranking duties as a condition sine qua non of rights. The contacts-based approach of the ICP can thus be seen as an opportunity to break this vicious circle, by bridging the tension between the MCP and the NCP. This position of an arbiter must be taken seriously, since it can reorient the focus of both policy paradigms and invite them to centre their views on contact between people from different backgrounds, including nationals. We can perfectly consider that both the MCP and the NCP are diversity strategies to ensure the necessary conditions for contact. Multiculturalism’s concern about equality and power sharing and the civic integrationist concern about minimum standards of language and knowledge on tradition and values are contributing ultimately to the promotion of “contact zones”. But the contacts-based approach is not consubstantial to the other two policy paradigms. This follows the need to abandon this old-fashioned universalist view of diversity policies as being a single comprehensive and integral way to manage the Unity and Diversity nexus. In an ideal distribution of functions, the MCP would be in charge of protecting diversity while the NCP would be tasked with defending unity (however this Unity needs to be rebooted incoporating diversity). The contacts-based approach of the ICP cannot be successful if a minimum (but limited) range of multicultural and national civic conditions are not met. But the fact that MCP and NCP are able to create the conditions for contact does not necessarily mean that it will happen. Consequently, there is a need for a policy the main target of which is to encourage contact among people. It is here that we can find interculturalism’s main scope for legitimization. The golden rule of intercultural citizenship (Zapata-Barrero, 2016b) is that through contacts people socialize in diversity and develop feelings of membership. There cannot be integration and inclusion without contact with other people with different backgrounds. It is through contact that people develop trust and solidarity, it is through contact that people develop a new public culture, a *culture of diversity*. Taking this building block of the ICP, in the current research agenda we are now in the phase of looking for factors to consolidate the policy paradigm, as markers of European identity (Zapata-Barrero, 2017a).

There are of course many variables that could be considered to ensure the consolidation of a policy paradigm. At the moment, the internal intercultural debate has been centred (probably because of its origins) on the focusing of economic benefits. That is, to put it in layman’s terms, when the ICP becomes a comprehensive and integral city project, the benefits of diversity management through contact promotion are empirically tested at the economic level. But there is still not a specific link that has been tested at the social level. In my view, this is what needs to be done now. This basically means incorporating the xenophobia-reduction and the solidarity-promotion hypotheses. This involves a decline in racism together with a reduction of negative public opinions towards immigrants, a reduction of the space for populist narratives (Zapata-Barrero, 2011) and even the prevention of rises in radicalism. In positive terms, the ICP could be seen as a policy favouring not only community cohesion but even more, national solidarity, and can reduce the “corroding effects” of diversity, especially in contexts where diversity can be seen as countering liberal values. In this sense, we can say that the ICP’s main challenge today is how it can find new forms of collective solidarity beyond class, nation and race.

Seen through a European lens, the current process by which the re-nationalization of policies, xenophobia, racism and intolerance are becoming a new “political ideology” has them framing public opinion and political discourses, and legitimizing policies (Zapata-Barrero & Triandafyllidou, 2012; Triandafyllidou et al., 2011). Scholarly work highlights that while this originates in cultural anxiety, it also emerges from approaches to welfare chauvinism, entrenched inequalities and emerging insecurity, all of which are also nurtured by the inconsistencies arising from the management of diversity and complex issues such as access to European territory.

Populism and neo-conservatism are the main forms that this new ideology takes. Most of the public debate around migration and diversity is essentially focused either on the explanatory level, seeking to identify the main factors provoking such an emergence, or on the strategies seeking to invade political power and governments, but much less so on the political and policy instruments we have for preventing and reducing the conditions that make it possible. This is where the ICP can play a central role.

The pressing situation today is clear: there is a lack of support for diversity management in the current post-M atmosphere. The new context of superdiversity and transnationalism together with the embracing of radicalization by second generation migrants poses a very volatile situation for Europe. The latest European Commission against Racism and Intolerance (ECRI) report, for instance, signals a growing anti-immigrant sentiment and Islamophobia as being among the key trends in 2015 (European Commission against Racism and Intolerance (ECRI), 2016). The recent terrorist attacks in Paris, Copenhagen, Nice and Berlin further add to the Islamophobic sentiment being misused by populist political parties to stir up prejudice and hatred against Muslims in general. Likewise, the decision taken in June 2016 by the UK to leave the European Union (Brexit) is also connected to anti-immigrant sentiments. Key questions arise today that cannot be answered with old policy paradigms: Can the MCP narrative counter the extremist narrative and/or the nationalist narrative? Can the MCP today be a marker of European identity without creating more political cleavages at the national level?

At the same time, in most EU and Council of Europe documents, interculturalism is linked to European values such as human rights, democracy and a culture of peace and dialogue, and to European identity (European Commission, 2008; Council of Europe, 2008; Vidmar-Horvat, 2012; Bekemans, 2012). This view of diversity as constitutive of the new European identity signals the fact that the latter is neither a pre-existing quality nor a historical given, but rather a process in the making, an identity to be achieved (Bauman, 2004; Bruter, 2005). Together with other inter-governmental institutions bringing together civil society and citizens across the Mediterranean, the Anna Lindh Foundation’s 10-year strategy “Working Together Towards 2025” (Anna Lindh Foundation, 2015) also argues for interculturalism as an alternative to the extremist narrative.

This particular function of the ICP has still not been examined both theoretically and empirically, and could be analysed at different levels. From a political party point of view, the xenophobia-reduction and the solidarity-promotion hypotheses can mean that the application of the ICP tends to leave no place for political parties with clear nationalist, xenophobic narratives and can promote mutual understanding and respect as well as new forms of living together and solidarity. So probably the main argument that can consolidate the ICP, so that it can become a mainstreaming policy, is that it can contribute to reducing the two main drivers of the xenophobic narrative: an increase in specific policies for a) a privileged culturally differentiated group of people and b) public expenditure.

We know that empirical studies are full of not only theoretical assumptions but theoretical thoughts about empirical assumptions. How to reconcile theoretical and empirical thinking is a crucial methodological challenge for scientific innovation and a real imperative to influence societal processes of change and political decisions in Europe. It is in this scholarly context that I set the challenge to consolidate the emerging ICP, taking into account the Unity/Diversity framework debate with the MCP and the NCP. However, there also remains work to be done on the nexus between xenophobia-reduction, solidarity-promotion and interculturalism. This would certainly address the gap between theoretical assumptions and policy outcomes, something the MCP does not appear to have achieved.

## Abbreviations

ICP: Intercultural policy paradigm
MCP: Multicultural policy paradigm
NCP: National civic policy paradigm
Post-M: Post-multicultural

## Footnotes

1 See Joppke (2004, 2007); Bauböck and Joppke (2010); Goodman (2012); Meer et al. (2015); Mouritsen (2008); Mouritsen (2011); Zapata-Barrero (2009).

2 The anti-racist dimension of interculturalism has been examined in depth by Gundara (2000, 2005). Only recently it has also been applied in policy studies (Pinxten, Cornelis, & Rubinstein, 2007; Ravinder, 2012; Carr, 2016).

3 I will take some works debating the policy narrative frameworks (Jones & McBeth, 2010; Shanahan, Mcbeth, & Hathaway, 2011; McBeth, Clemons, Husmann, Kusko, & Gaarden, 2013) and others following an interesting emerging debate on policy paradigm change (Campbell, 2002; Hogan & Howlett, 2015) taking as reference the path-breaking work of Hall (1993). I have developed some of this in more detail in Zapata-Barrero (2017a).

4 On the concept of multiple diversity, applied in the case of Spain, see Zapata-Barrero (2013).

5 See Borkert, Bosswick, Heckmann, and Lüken-Klaben (2007); Lüken-Klaßen and Heckmann (2010); Caponio and Borkert (2010); and Caponio and Ricucci (2015). This last work provides an overview as to how the debate was introduced by EU institutions.

6 The concept of diversity advantage has been introduced by the UK think tank Comedia (2017), directed by Wood (2004. See also Council of Europe, 2008) and mainly inspired by Zachary’s (2003) seminal work. Here we can also mention the seminal Unesco document (1996), considering diversity as a good resource in need of protection, even forming part of our European cultural heritage and the basis of Agenda 21 for culture, taking diversity’s potential for creativity as the main diversity advantage.

7 These power relations between people and socio-economical contrasts are fully addressed in the work of Cantle (2012) for instance. It is an area that certainly needs deeper examination.
